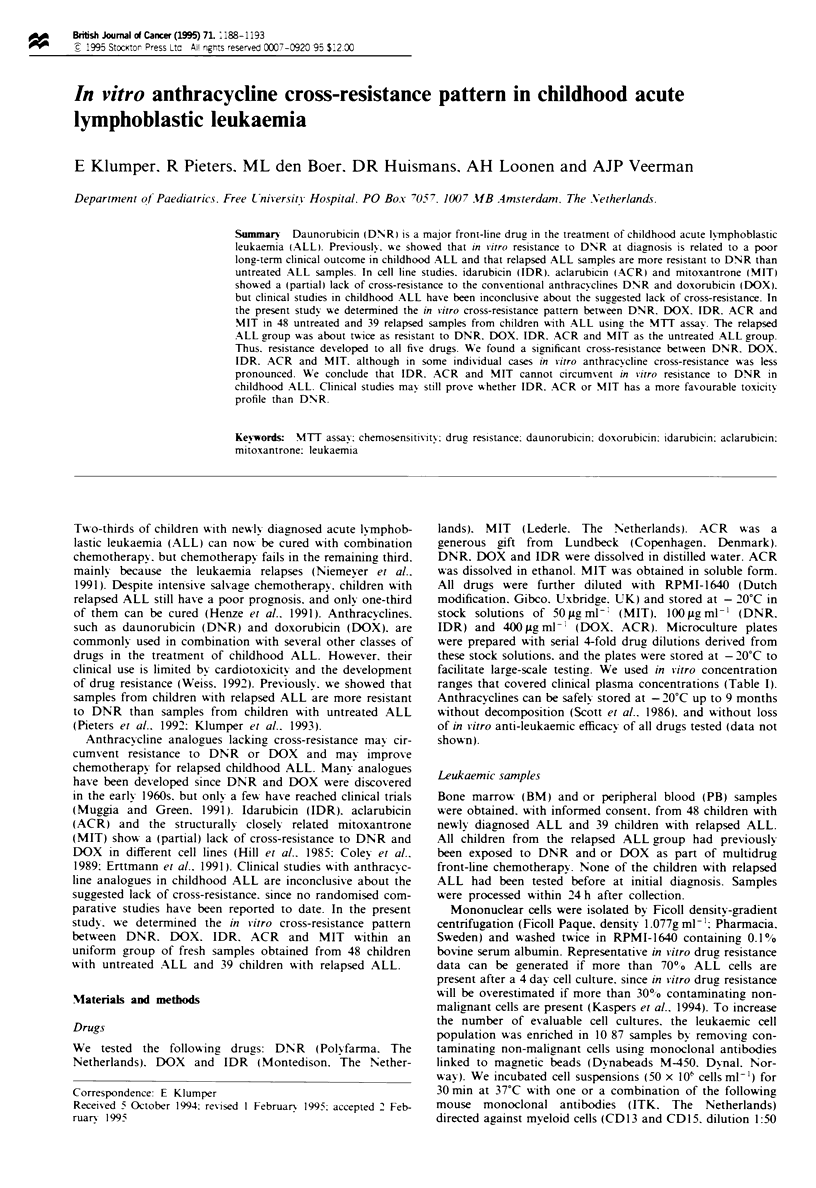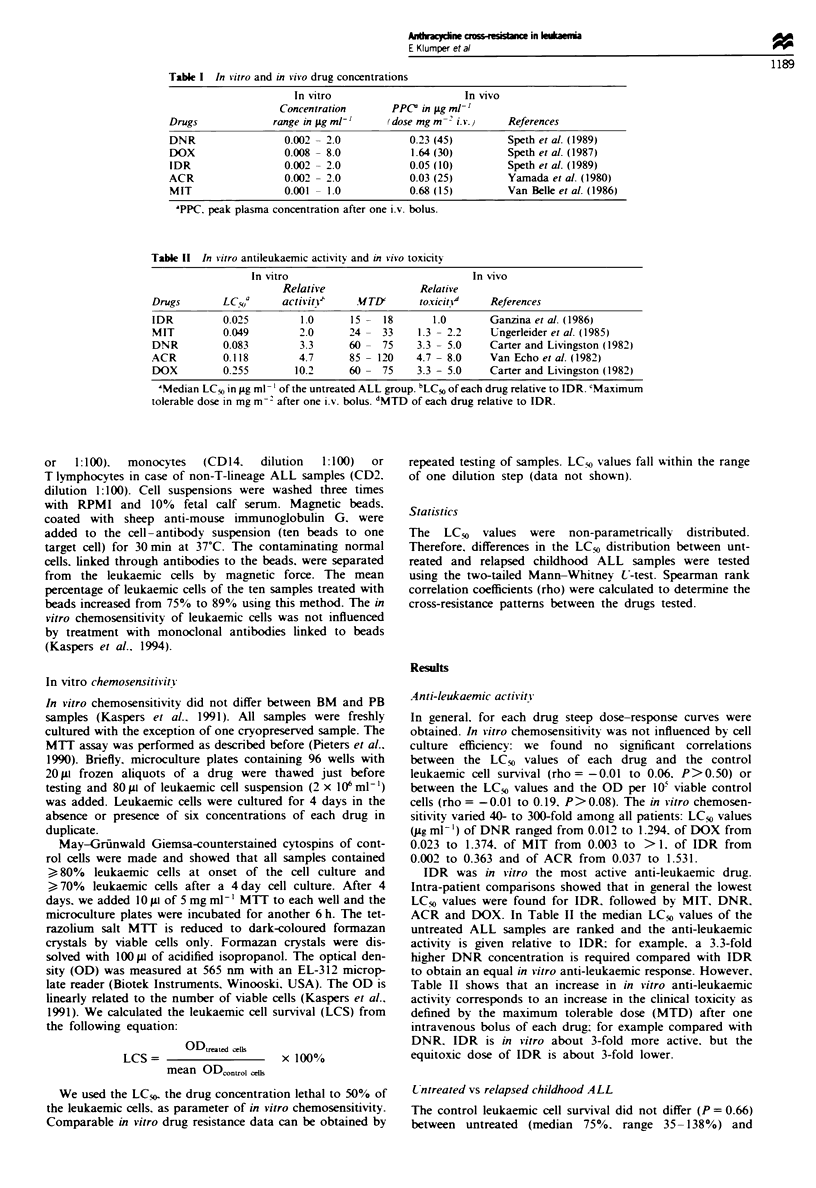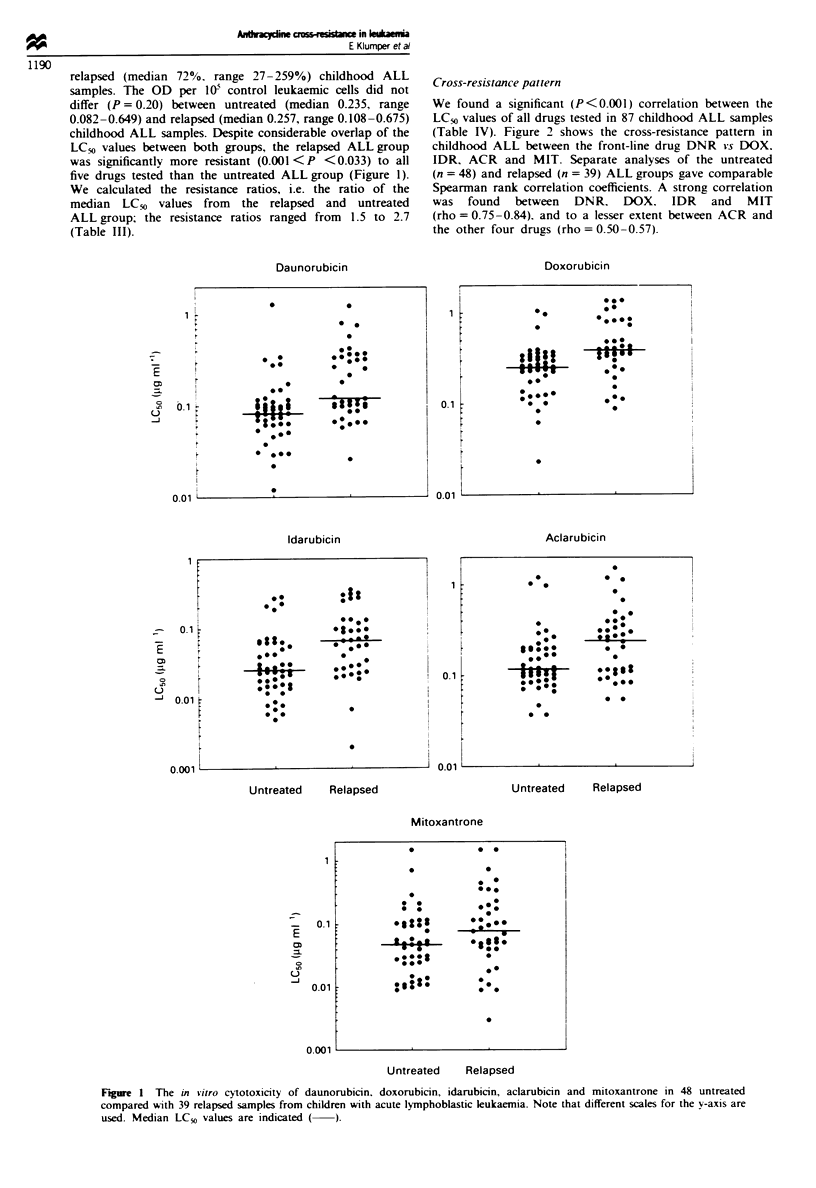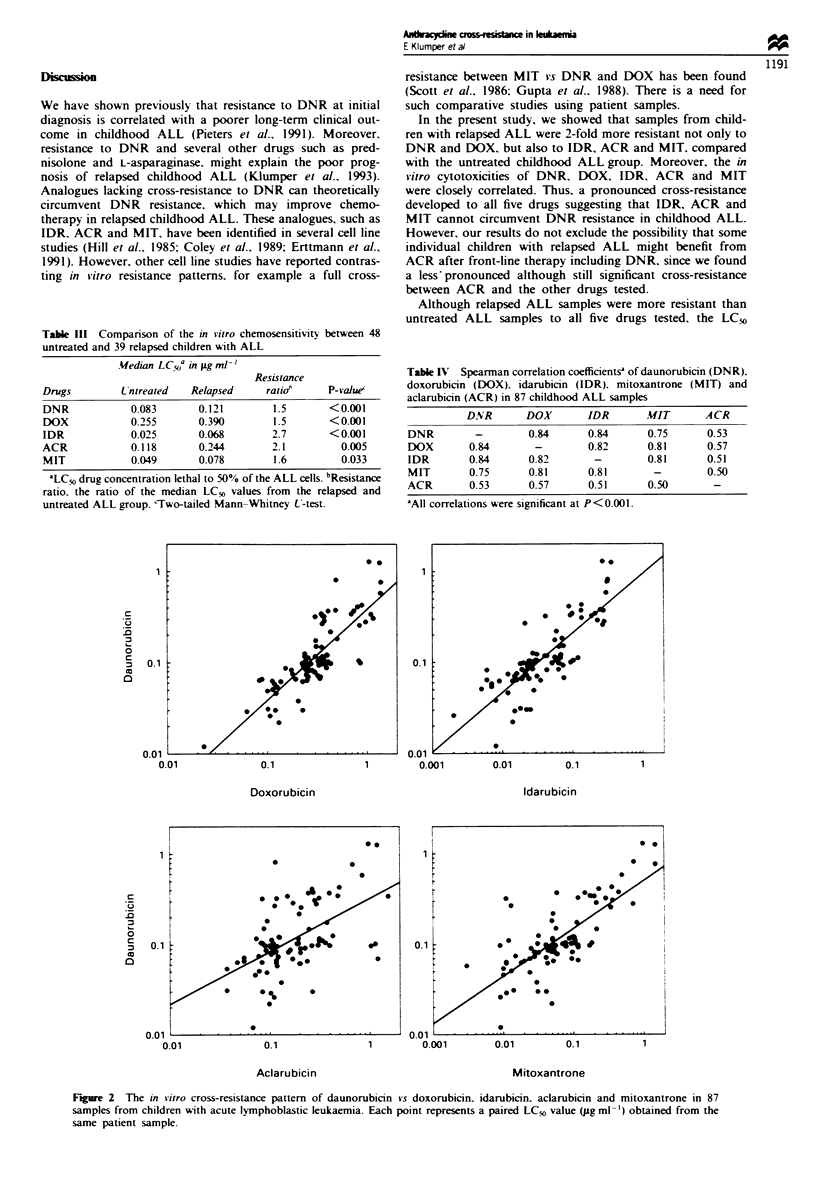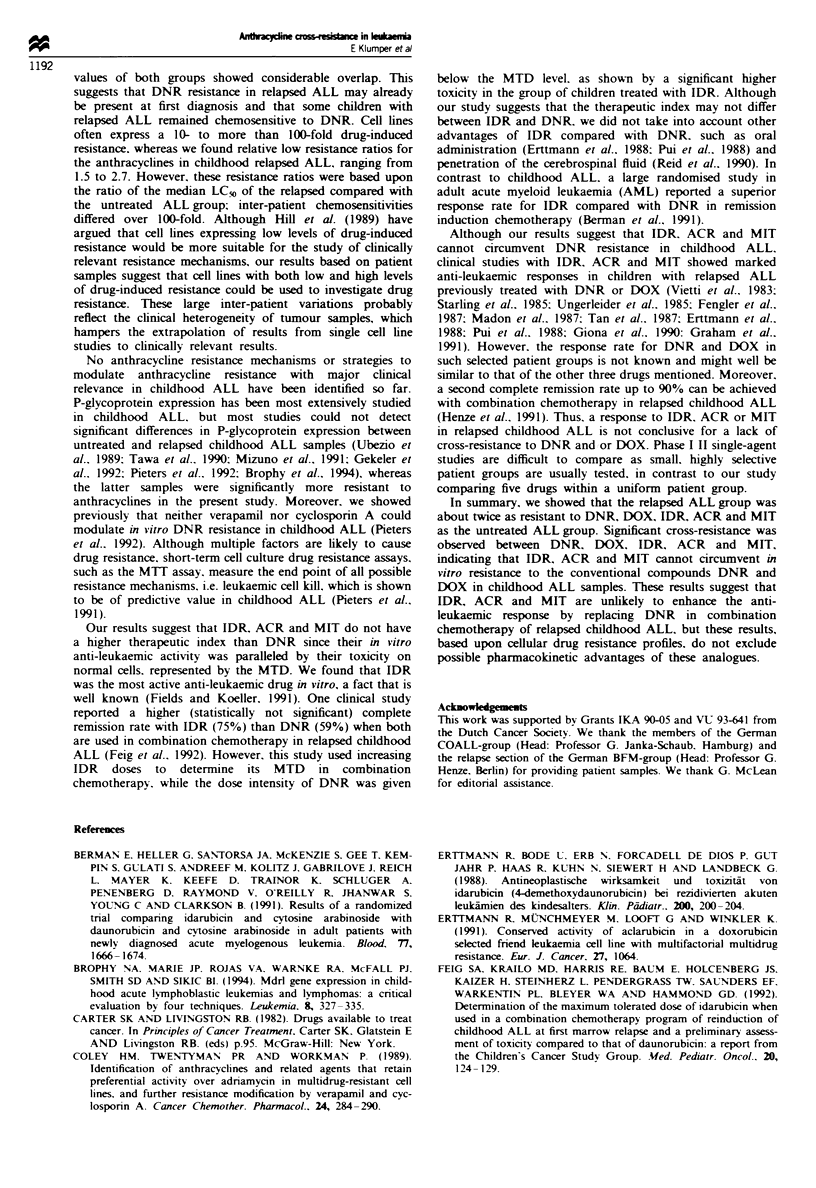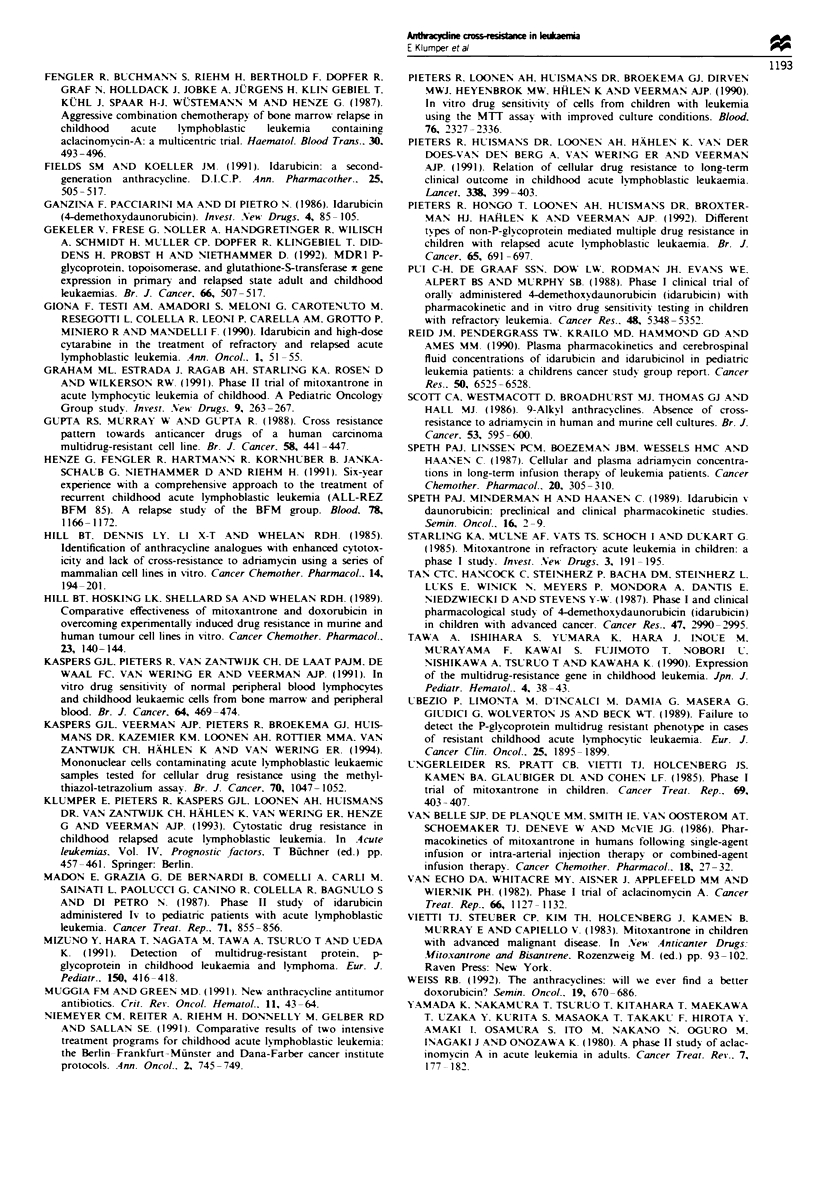# In vitro anthracycline cross-resistance pattern in childhood acute lymphoblastic leukaemia.

**DOI:** 10.1038/bjc.1995.231

**Published:** 1995-06

**Authors:** E. Klumper, R. Pieters, M. L. den Boer, D. R. Huismans, A. H. Loonen, A. J. Veerman

**Affiliations:** Department of Paediatrics, Free University Hospital, Amsterdam, The Netherlands.

## Abstract

Daunorubicin (DNR) is a major front-line drug in the treatment of childhood acute lymphoblastic leukaemia (ALL). Previously, we showed that in vitro resistance to DNR at diagnosis is related to a poor long-term clinical outcome in childhood ALL and that relapsed ALL samples are more resistant to DNR than untreated ALL samples. In cell line studies, idarubicin (IDR), aclarubicin (ACR) and mitoxantrone (MIT) showed a (partial) lack of cross-resistance to the conventional anthracyclines DNR and doxorubicin (DOX), but clinical studies in childhood ALL have been inconclusive about the suggested lack of cross-resistance. In the present study we determined the in vitro cross-resistance pattern between DNR, DOX, IDR, ACR and MIT in 48 untreated and 39 relapsed samples from children with ALL using the MTT assay. The relapsed ALL group was about twice as resistant to DNR, DOX, IDR, ACR and MTT as the untreated ALL group. Thus, resistance developed to all five drugs. We found a significant cross-resistance between DNR, DOX, IDR, ACR and MIT, although in some individual cases in vitro anthracycline cross-resistance was less pronounced. We conclude that IDR, ACR and MIT cannot circumvent in vitro resistance to DNR in childhood ALL. Clinical studies may still prove whether IDR, ACR or MIT has a more favourable toxicity profile than DNR.


British Journal of Cancer (1995) 71, 1188-1193

© 1995 Stockton Press Ltd All rights reserved 0007-0920/95 $12.00

### *In vitro* anthracycline cross-resistance pattern in childhood acute lymphoblastic leukaemia

E Klumper, R Pieters, ML den Boer, DR Huismans, AH Loonen and AJP Veerman

*Department of Paediatrics, Free University Hospital, PO Box 7057, 1007 MB Amsterdam, The Netherlands.*

**Summary** Daunorubicin (DNR) is a major front-line drug in the treatment of childhood acute lymphoblastic leukaemia (ALL). Previously, we showed that *in vitro* resistance to DNR at diagnosis is related to a poor long-term clinical outcome in childhood ALL and that relapsed ALL samples are more resistant to DNR than untreated ALL samples. In cell line studies, idarubicin (IDR), aclarubicin (ACR) and mitoxantrone (MIT) showed a (partial) lack of cross-resistance to the conventional anthracyclines DNR and doxorubicin (DOX), but clinical studies in childhood ALL have been inconclusive about the suggested lack of cross-resistance. In the present study we determined the *in vitro* cross-resistance pattern between DNR, DOX, IDR, ACR and MIT in 48 untreated and 39 relapsed samples from children with ALL using the MTT assay. The relapsed ALL group was about twice as resistant to DNR, DOX, IDR, ACR and MIT as the untreated ALL group. Thus, resistance developed to all five drugs. We found a significant cross-resistance between DNR, DOX, IDR, ACR and MIT, although in some individual cases *in vitro* anthracycline cross-resistance was less pronounced. We conclude that IDR, ACR and MIT cannot circumvent *in vitro* resistance to DNR in childhood ALL. Clinical studies may still prove whether IDR, ACR or MIT has a more favourable toxicity profile than DNR.

**Keywords:** MTT assay; chemosensitivity; drug resistance; daunorubicin; doxorubicin; idarubicin; aclarubicin; mitoxantrone; leukaemia

Two-thirds of children with newly diagnosed acute lymphoblastic leukaemia (ALL) can now be cured with combination chemotherapy, but chemotherapy fails in the remaining third, mainly because the leukaemia relapses (Niemeyer *et al*., 1991). Despite intensive salvage chemotherapy, children with relapsed ALL still have a poor prognosis, and only one-third of them can be cured (Henze *et al*., 1991). Anthracyclines, such as daunorubicin (DNR) and doxorubicin (DOX), are commonly used in combination with several other classes of drugs in the treatment of childhood ALL. However, their clinical use is limited by cardiotoxicity and the development of drug resistance (Weiss, 1992). Previously, we showed that samples from children with relapsed ALL are more resistant to DNR than samples from children with untreated ALL (Pieters *et al*., 1992; Klumper *et al*., 1993).

Anthracycline analogues lacking cross-resistance may circumvent resistance to DNR or DOX and may improve chemotherapy for relapsed childhood ALL. Many analogues have been developed since DNR and DOX were discovered in the early 1960s, but only a few have reached clinical trials (Muggia and Green, 1991). Idarubicin (IDR), aclarubicin (ACR) and the structurally closely related mitoxantrone (MIT) show a (partial) lack of cross-resistance to DNR and DOX in different cell lines (Hill *et al*., 1985; Coley *et al*., 1989; Erttmann *et al*., 1991). Clinical studies with anthracycline analogues in childhood ALL are inconclusive about the suggested lack of cross-resistance, since no randomised comparative studies have been reported to date. In the present study, we determined the *in vitro* cross-resistance pattern between DNR, DOX, IDR, ACR and MIT within an uniform group of fresh samples obtained from 48 children with untreated ALL and 39 children with relapsed ALL.

### Materials and methods

### *Drugs*

We tested the following drugs: DNR (Polyfarma, The Netherlands), DOX and IDR (Montedison, The Netherlands), MIT (Lederle, The Netherlands). ACR was a generous gift from Lundbeck (Copenhagen, Denmark). DNR, DOX and IDR were dissolved in distilled water. ACR was dissolved in ethanol. MIT was obtained in soluble form. All drugs were further diluted with RPMI-1640 (Dutch modification, Gibco, Uxbridge, UK) and stored at $-20°C$ in stock solutions of $50 \mu g ml^{-1}$ (MIT), $100 \mu g ml^{-1}$ (DNR, IDR) and $400 \mu g ml^{-1}$ (DOX, ACR). Microculture plates were prepared with serial 4-fold drug dilutions derived from these stock solutions, and the plates were stored at $-20°C$ to facilitate large-scale testing. We used *in vitro* concentration ranges that covered clinical plasma concentrations (Table I). Anthracyclines can be safely stored at $-20°C$ up to 9 months without decomposition (Scott *et al*., 1986), and without loss of *in vitro* anti-leukaemic efficacy of all drugs tested (data not shown).

### *Leukaemic samples*

Bone marrow (BM) and/or peripheral blood (PB) samples were obtained, with informed consent, from 48 children with newly diagnosed ALL and 39 children with relapsed ALL. All children from the relapsed ALL group had previously been exposed to DNR and/or DOX as part of multidrug front-line chemotherapy. None of the children with relapsed ALL had been tested before at initial diagnosis. Samples were processed within 24 h after collection.

Mononuclear cells were isolated by Ficoll density-gradient centrifugation (Ficoll Paque, density $1.077 g ml^{-1}$; Pharmacia, Sweden) and washed twice in RPMI-1640 containing 0.1% bovine serum albumin. Representative *in vitro* drug resistance data can be generated if more than 70% ALL cells are present after a 4 day cell culture, since *in vitro* drug resistance will be overestimated if more than 30% contaminating non-malignant cells are present (Kaspers *et al*., 1994). To increase the number of evaluable cell cultures, the leukaemic cell population was enriched in 10/87 samples by removing contaminating non-malignant cells using monoclonal antibodies linked to magnetic beads (Dynabeads M-450, Dynal, Norway). We incubated cell suspensions ($50 \times 10^6$ cells $ml^{-1}$) for 30 min at 37°C with one or a combination of the following mouse monoclonal antibodies (ITK, The Netherlands) directed against myeloid cells (CD13 and CD15, dilution 1:50

Correspondence: E Klumper
Received 5 October 1994; revised 1 February 1995; accepted 2 February 1995

**Table I** *In vitro* and *in vivo* drug concentrations

| | In vitro | In vivo | |
|---|---|---|---|
| *Drugs* | *Concentration range in μg ml⁻¹* | *PPC[a] in μg ml⁻¹ (dose mg m⁻² i.v.)* | *References* |
| DNR | 0.002 - 2.0 | 0.23 (45) | Speth *et al.* (1989) |
| DOX | 0.008 - 8.0 | 1.64 (30) | Speth *et al.* (1987) |
| IDR | 0.002 - 2.0 | 0.05 (10) | Speth *et al.* (1989) |
| ACR | 0.002 - 2.0 | 0.03 (25) | Yamada *et al.* (1980) |
| MIT | 0.001 - 1.0 | 0.68 (15) | Van Belle *et al.* (1986) |

[a]PPC, peak plasma concentration after one i.v. bolus.

**Table II** *In vitro* antileukaemic activity and *in vivo* toxicity

| | In vitro | | In vivo | | |
|---|---|---|---|---|---|
| *Drugs* | $LC_{50}$[a] | *Relative activity*[b] | *MTD*[c] | *Relative toxicity*[d] | *References* |
| IDR | 0.025 | 1.0 | 15 - 18 | 1.0 | Ganzina *et al.* (1986) |
| MIT | 0.049 | 2.0 | 24 - 33 | 1.3 - 2.2 | Ungerleider *et al.* (1985) |
| DNR | 0.083 | 3.3 | 60 - 75 | 3.3 - 5.0 | Carter and Livingston (1982) |
| ACR | 0.118 | 4.7 | 85 - 120 | 4.7 - 8.0 | Van Echo *et al.* (1982) |
| DOX | 0.255 | 10.2 | 60 - 75 | 3.3 - 5.0 | Carter and Livingston (1982) |

[a]Median $LC_{50}$ in μg ml⁻¹ of the untreated ALL group. [b]$LC_{50}$ of each drug relative to IDR. [c]Maximum tolerable dose in mg m⁻² after one i.v. bolus. [d]MTD of each drug relative to IDR.

or 1:100), monocytes (CD14, dilution 1:100) or T lymphocytes in case of non-T-lineage ALL samples (CD2, dilution 1:100). Cell suspensions were washed three times with RPMI and 10% fetal calf serum. Magnetic beads, coated with sheep anti-mouse immunoglobulin G, were added to the cell–antibody suspension (ten beads to one target cell) for 30 min at 37°C. The contaminating normal cells, linked through antibodies to the beads, were separated from the leukaemic cells by magnetic force. The mean percentage of leukaemic cells of the ten samples treated with beads increased from 75% to 89% using this method. The *in vitro* chemosensitivity of leukaemic cells was not influenced by treatment with monoclonal antibodies linked to beads (Kaspers *et al.*, 1994).

### In vitro *chemosensitivity*

*In vitro* chemosensitivity did not differ between BM and PB samples (Kaspers *et al.*, 1991). All samples were freshly cultured with the exception of one cryopreserved sample. The MTT assay was performed as described before (Pieters *et al.*, 1990). Briefly, microculture plates containing 96 wells with 20 μl frozen aliquots of a drug were thawed just before testing and 80 μl of leukaemic cell suspension ($2 \times 10^6$ ml⁻¹) was added. Leukaemic cells were cultured for 4 days in the absence or presence of six concentrations of each drug in duplicate.

May–Grünwald Giemsa-counterstained cytospins of control cells were made and showed that all samples contained ⩾80% leukaemic cells at onset of the cell culture and ⩾70% leukaemic cells after a 4 day cell culture. After 4 days, we added 10 μl of 5 mg ml⁻¹ MTT to each well and the microculture plates were incubated for another 6 h. The tetrazolium salt MTT is reduced to dark-coloured formazan crystals by viable cells only. Formazan crystals were dissolved with 100 μl of acidified isopropanol. The optical density (OD) was measured at 565 nm with an EL-312 microplate reader (Biotek Instruments, Winooski, USA). The OD is linearly related to the number of viable cells (Kaspers *et al.*, 1991). We calculated the leukaemic cell survival (LCS) from the following equation:

$$LCS = \frac{OD_{\text{treated cells}}}{\text{mean } OD_{\text{control cells}}} \times 100\%$$

We used the $LC_{50}$, the drug concentration lethal to 50% of the leukaemic cells, as parameter of *in vitro* chemosensitivity. Comparable *in vitro* drug resistance data can be obtained by repeated testing of samples. $LC_{50}$ values fall within the range of one dilution step (data not shown).

### *Statistics*

The $LC_{50}$ values were non-parametrically distributed. Therefore, differences in the $LC_{50}$ distribution between untreated and relapsed childhood ALL samples were tested using the two-tailed Mann–Whitney *U*-test. Spearman rank correlation coefficients (rho) were calculated to determine the cross-resistance patterns between the drugs tested.

### Results

### *Anti-leukaemic activity*

In general, for each drug steep dose–response curves were obtained. *In vitro* chemosensitivity was not influenced by cell culture efficiency: we found no significant correlations between the $LC_{50}$ values of each drug and the control leukaemic cell survival (rho = −0.01 to 0.06, $P > 0.50$) or between the $LC_{50}$ values and the OD per $10^5$ viable control cells (rho = −0.01 to 0.19, $P > 0.08$). The *in vitro* chemosensitivity varied 40- to 300-fold among all patients: $LC_{50}$ values (μg ml⁻¹) of DNR ranged from 0.012 to 1.294, of DOX from 0.023 to 1.374, of MIT from 0.003 to >1, of IDR from 0.002 to 0.363 and of ACR from 0.037 to 1.531.

IDR was *in vitro* the most active anti-leukaemic drug. Intra-patient comparisons showed that in general the lowest $LC_{50}$ values were found for IDR, followed by MIT, DNR, ACR and DOX. In Table II the median $LC_{50}$ values of the untreated ALL samples are ranked and the anti-leukaemic activity is given relative to IDR; for example, a 3.3-fold higher DNR concentration is required compared with IDR to obtain an equal *in vitro* anti-leukaemic response. However, Table II shows that an increase in *in vitro* anti-leukaemic activity corresponds to an increase in the clinical toxicity as defined by the maximum tolerable dose (MTD) after one intravenous bolus of each drug; for example compared with DNR, IDR is *in vitro* about 3-fold more active, but the equitoxic dose of IDR is about 3-fold lower.

### *Untreated* vs *relapsed childhood ALL*

The control leukaemic cell survival did not differ ($P = 0.66$) between untreated (median 75%, range 35–138%) and relapsed (median 72%, range 27–259%) childhood ALL samples. The OD per $10^5$ control leukaemic cells did not differ ($P = 0.20$) between untreated (median 0.235, range 0.082–0.649) and relapsed (median 0.257, range 0.108–0.675) childhood ALL samples. Despite considerable overlap of the $LC_{50}$ values between both groups, the relapsed ALL group was significantly more resistant ($0.001 < P < 0.033$) to all five drugs tested than the untreated ALL group (Figure 1). We calculated the resistance ratios, i.e. the ratio of the median $LC_{50}$ values from the relapsed and untreated ALL group; the resistance ratios ranged from 1.5 to 2.7 (Table III).

*Cross-resistance pattern*

We found a significant ($P < 0.001$) correlation between the $LC_{50}$ values of all drugs tested in 87 childhood ALL samples (Table IV). Figure 2 shows the cross-resistance pattern in childhood ALL between the front-line drug DNR *vs* DOX, IDR, ACR and MIT. Separate analyses of the untreated ($n = 48$) and relapsed ($n = 39$) ALL groups gave comparable Spearman rank correlation coefficients. A strong correlation was found between DNR, DOX, IDR and MIT (rho = 0.75–0.84), and to a lesser extent between ACR and the other four drugs (rho = 0.50–0.57).

**Figure 1** The *in vitro* cytotoxicity of daunorubicin, doxorubicin, idarubicin, aclarubicin and mitoxantrone in 48 untreated compared with 39 relapsed samples from children with acute lymphoblastic leukaemia. Note that different scales for the y-axis are used. Median $LC_{50}$ values are indicated (——).

### Discussion

We have shown previously that resistance to DNR at initial diagnosis is correlated with a poorer long-term clinical outcome in childhood ALL (Pieters *et al.*, 1991). Moreover, resistance to DNR and several other drugs such as prednisolone and L-asparaginase, might explain the poor prognosis of relapsed childhood ALL (Klumper *et al.*, 1993). Analogues lacking cross-resistance to DNR can theoretically circumvent DNR resistance, which may improve chemotherapy in relapsed childhood ALL. These analogues, such as IDR, ACR and MIT, have been identified in several cell line studies (Hill *et al.*, 1985; Coley *et al.*, 1989; Erttmann *et al.*, 1991). However, other cell line studies have reported contrasting *in vitro* resistance patterns, for example a full cross-resistance between MIT *vs* DNR and DOX has been found (Scott *et al.*, 1986; Gupta *et al.*, 1988). There is a need for such comparative studies using patient samples.

In the present study, we showed that samples from children with relapsed ALL were 2-fold more resistant not only to DNR and DOX, but also to IDR, ACR and MIT, compared with the untreated childhood ALL group. Moreover, the *in vitro* cytotoxicities of DNR, DOX, IDR, ACR and MIT were closely correlated. Thus, a pronounced cross-resistance developed to all five drugs suggesting that IDR, ACR and MIT cannot circumvent DNR resistance in childhood ALL. However, our results do not exclude the possibility that some individual children with relapsed ALL might benefit from ACR after front-line therapy including DNR, since we found a less pronounced although still significant cross-resistance between ACR and the other drugs tested.

Although relapsed ALL samples were more resistant than untreated ALL samples to all five drugs tested, the $LC_{50}$

**Table III** Comparison of the *in vitro* chemosensitivity between 48 untreated and 39 relapsed children with ALL

| Drugs | Median $LC_{50}$[a] in μg ml$^{-1}$ Untreated | Relapsed | Resistance ratio[b] | P-value[c] |
|---|---|---|---|---|
| DNR | 0.083 | 0.121 | 1.5 | <0.001 |
| DOX | 0.255 | 0.390 | 1.5 | <0.001 |
| IDR | 0.025 | 0.068 | 2.7 | <0.001 |
| ACR | 0.118 | 0.244 | 2.1 | 0.005 |
| MIT | 0.049 | 0.078 | 1.6 | 0.033 |

[a]$LC_{50}$ drug concentration lethal to 50% of the ALL cells. [b]Resistance ratio, the ratio of the median $LC_{50}$ values from the relapsed and untreated ALL group. [c]Two-tailed Mann–Whitney *U*-test.

**Table IV** Spearman correlation coefficients[a] of daunorubicin (DNR), doxorubicin (DOX), idarubicin (IDR), mitoxantrone (MIT) and aclarubicin (ACR) in 87 childhood ALL samples

| | *DNR* | *DOX* | *IDR* | *MIT* | *ACR* |
|---|---|---|---|---|---|
| DNR | – | 0.84 | 0.84 | 0.75 | 0.53 |
| DOX | 0.84 | – | 0.82 | 0.81 | 0.57 |
| IDR | 0.84 | 0.82 | – | 0.81 | 0.51 |
| MIT | 0.75 | 0.81 | 0.81 | – | 0.50 |
| ACR | 0.53 | 0.57 | 0.51 | 0.50 | – |

[a]All correlations were significant at $P < 0.001$.

**Figure 2** The *in vitro* cross-resistance pattern of daunorubicin *vs* doxorubicin, idarubicin, aclarubicin and mitoxantrone in 87 samples from children with acute lymphoblastic leukaemia. Each point represents a paired $LC_{50}$ value (μg ml$^{-1}$) obtained from the same patient sample.

values of both groups showed considerable overlap. This suggests that DNR resistance in relapsed ALL may already be present at first diagnosis and that some children with relapsed ALL remained chemosensitive to DNR. Cell lines often express a 10- to more than 100-fold drug-induced resistance, whereas we found relative low resistance ratios for the anthracyclines in childhood relapsed ALL, ranging from 1.5 to 2.7. However, these resistance ratios were based upon the ratio of the median $LC_{50}$ of the relapsed compared with the untreated ALL group; inter-patient chemosensitivities differed over 100-fold. Although Hill *et al.* (1989) have argued that cell lines expressing low levels of drug-induced resistance would be more suitable for the study of clinically relevant resistance mechanisms, our results based on patient samples suggest that cell lines with both low and high levels of drug-induced resistance could be used to investigate drug resistance. These large inter-patient variations probably reflect the clinical heterogeneity of tumour samples, which hampers the extrapolation of results from single cell line studies to clinically relevant results.

No anthracycline resistance mechanisms or strategies to modulate anthracycline resistance with major clinical relevance in childhood ALL have been identified so far. P-glycoprotein expression has been most extensively studied in childhood ALL, but most studies could not detect significant differences in P-glycoprotein expression between untreated and relapsed childhood ALL samples (Ubezio *et al.*, 1989; Tawa *et al.*, 1990; Mizuno *et al.*, 1991; Gekeler *et al.*, 1992; Pieters *et al.*, 1992; Brophy *et al.*, 1994), whereas the latter samples were significantly more resistant to anthracyclines in the present study. Moreover, we showed previously that neither verapamil nor cyclosporin A could modulate *in vitro* DNR resistance in childhood ALL (Pieters *et al.*, 1992). Although multiple factors are likely to cause drug resistance, short-term cell culture drug resistance assays, such as the MTT assay, measure the end point of all possible resistance mechanisms, i.e. leukaemic cell kill, which is shown to be of predictive value in childhood ALL (Pieters *et al.*, 1991).

Our results suggest that IDR, ACR and MIT do not have a higher therapeutic index than DNR since their *in vitro* anti-leukaemic activity was paralleled by their toxicity on normal cells, represented by the MTD. We found that IDR was the most active anti-leukaemic drug *in vitro*, a fact that is well known (Fields and Koeller, 1991). One clinical study reported a higher (statistically not significant) complete remission rate with IDR (75%) than DNR (59%) when both are used in combination chemotherapy in relapsed childhood ALL (Feig *et al.*, 1992). However, this study used increasing IDR doses to determine its MTD in combination chemotherapy, while the dose intensity of DNR was given below the MTD level, as shown by a significant higher toxicity in the group of children treated with IDR. Although our study suggests that the therapeutic index may not differ between IDR and DNR, we did not take into account other advantages of IDR compared with DNR, such as oral administration (Erttmann *et al.*, 1988; Pui *et al.*, 1988) and penetration of the cerebrospinal fluid (Reid *et al.*, 1990). In contrast to childhood ALL, a large randomised study in adult acute myeloid leukaemia (AML) reported a superior response rate for IDR compared with DNR in remission induction chemotherapy (Berman *et al.*, 1991).

Although our results suggest that IDR, ACR and MIT cannot circumvent DNR resistance in childhood ALL, clinical studies with IDR, ACR and MIT showed marked anti-leukaemic responses in children with relapsed ALL previously treated with DNR or DOX (Vietti *et al.*, 1983; Starling *et al.*, 1985; Ungerleider *et al.*, 1985; Fengler *et al.*, 1987; Madon *et al.*, 1987; Tan *et al.*, 1987; Erttmann *et al.*, 1988; Pui *et al.*, 1988; Giona *et al.*, 1990; Graham *et al.*, 1991). However, the response rate for DNR and DOX in such selected patient groups is not known and might well be similar to that of the other three drugs mentioned. Moreover, a second complete remission rate up to 90% can be achieved with combination chemotherapy in relapsed childhood ALL (Henze *et al.*, 1991). Thus, a response to IDR, ACR or MIT in relapsed childhood ALL is not conclusive for a lack of cross-resistance to DNR and/or DOX. Phase I/II single-agent studies are difficult to compare as small, highly selective patient groups are usually tested, in contrast to our study comparing five drugs within a uniform patient group.

In summary, we showed that the relapsed ALL group was about twice as resistant to DNR, DOX, IDR, ACR and MIT as the untreated ALL group. Significant cross-resistance was observed between DNR, DOX, IDR, ACR and MIT, indicating that IDR, ACR and MIT cannot circumvent *in vitro* resistance to the conventional compounds DNR and DOX in childhood ALL samples. These results suggest that IDR, ACR and MIT are unlikely to enhance the anti-leukaemic response by replacing DNR in combination chemotherapy of relapsed childhood ALL, but these results, based upon cellular drug resistance profiles, do not exclude possible pharmacokinetic advantages of these analogues.

**Acknowledgements**

This work was supported by Grants IKA 90-05 and VU 93-641 from the Dutch Cancer Society. We thank the members of the German COALL-group (Head: Professor G. Janka-Schaub, Hamburg) and the relapse section of the German BFM-group (Head: Professor G. Henze, Berlin) for providing patient samples. We thank G. McLean for editorial assistance.

**References**

BERMAN E, HELLER G, SANTORSA JA, McKENZIE S, GEE T, KEMPIN S, GULATI S, ANDREEF M, KOLITZ J, GABRILOVE J, REICH L, MAYER K, KEEFE D, TRAINOR K, SCHLUGER A, PENENBERG D, RAYMOND V, O'REILLY R, JHANWAR S, YOUNG C AND CLARKSON B. (1991). Results of a randomized trial comparing idarubicin and cytosine arabinoside with daunorubicin and cytosine arabinoside in adult patients with newly diagnosed acute myelogenous leukemia. *Blood*, **77**, 1666–1674.

BROPHY NA, MARIE JP, ROJAS VA, WARNKE RA, McFALL PJ, SMITH SD AND SIKIC BI. (1994). Mdrl gene expression in childhood acute lymphoblastic leukemias and lymphomas: a critical evaluation by four techniques. *Leukemia*, **8**, 327–335.

CARTER SK AND LIVINGSTON RB. (1982). Drugs available to treat cancer. In *Principles of Cancer Treatment*. Carter SK, Glatstein E AND Livingston RB. (eds) p.95. McGraw-Hill: New York.

COLEY HM, TWENTYMAN PR AND WORKMAN P. (1989). Identification of anthracyclines and related agents that retain preferential activity over adriamycin in multidrug-resistant cell lines, and further resistance modification by verapamil and cyclosporin A. *Cancer Chemother. Pharmacol.*, **24**, 284–290.

ERTTMANN R, BODE U, ERB N, FORCADELL DE DIOS P, GUTJAHR P, HAAS R, KUHN N, SIEWERT H AND LANDBECK G. (1988). Antineoplastische wirksamkeit und toxizität von idarubicin (4-demethoxydaunorubicin) bei rezidivierten akuten leukämien des kindesalters. *Klin. Pädiatr.*, **200**, 200–204.

ERTTMANN R, MÜNCHMEYER M, LOOFT G AND WINKLER K. (1991). Conserved activity of aclarubicin in a doxorubicin selected friend leukaemia cell line with multifactorial multidrug resistance. *Eur. J. Cancer*, **27**, 1064.

FEIG SA, KRAILO MD, HARRIS RE, BAUM E, HOLCENBERG JS, KAIZER H, STEINHERZ L, PENDERGRASS TW, SAUNDERS EF, WARKENTIN PL, BLEYER WA AND HAMMOND GD. (1992). Determination of the maximum tolerated dose of idarubicin when used in a combination chemotherapy program of reinduction of childhood ALL at first marrow relapse and a preliminary assessment of toxicity compared to that of daunorubicin: a report from the Children's Cancer Study Group. *Med. Pediatr. Oncol.*, **20**, 124–129.

FENGLER R. BUCHMANN S. RIEHM H. BERTHOLD F. DOPFER R. GRAF N. HOLLDACK J. JOBKE A. JÜRGENS H. KLIN GEBIEL T. KÜHL J. SPAAR H-J. WÜSTEMANN M AND HENZE G. (1987). Aggressive combination chemotherapy of bone marrow relapse in childhood acute lymphoblastic leukemia containing aclacinomycin-A: a multicentric trial. *Haematol. Blood Trans.*, **30**, 493-496.

FIELDS SM AND KOELLER JM. (1991). Idarubicin: a second-generation anthracycline. D.I.C.P. *Ann. Pharmacother.*, **25**, 505-517.

GANZINA F. PACCIARINI MA AND DI PIETRO N. (1986). Idarubicin (4-demethoxydaunorubicin). *Invest. New Drugs*, **4**, 85-105.

GEKELER V. FRESE G. NOLLER A. HANDGRETINGER R. WILISCH A. SCHMIDT H. MULLER CP. DOPFER R. KLINGEBIEL T. DIDDENS H. PROBST H AND NIETHAMMER D. (1992). MDR1 P-glycoprotein, topoisomerase, and glutathione-S-transferase π gene expression in primary and relapsed state adult and childhood leukaemias. *Br. J. Cancer*, **66**, 507-517.

GIONA F. TESTI AM. AMADORI S. MELONI G. CAROTENUTO M. RESEGOTTI L. COLELLA R. LEONI P. CARELLA AM. GROTTO P. MINIERO R AND MANDELLI F. (1990). Idarubicin and high-dose cytarabine in the treatment of refractory and relapsed acute lymphoblastic leukemia. *Ann. Oncol.*, **1**, 51-55.

GRAHAM ML. ESTRADA J. RAGAB AH. STARLING KA. ROSEN D AND WILKERSON RW. (1991). Phase II trial of mitoxantrone in acute lymphocytic leukemia of childhood. A Pediatric Oncology Group study. *Invest. New Drugs*, **9**, 263-267.

GUPTA RS. MURRAY W AND GUPTA R. (1988). Cross resistance pattern towards anticancer drugs of a human carcinoma multidrug-resistant cell line. *Br. J. Cancer*, **58**, 441-447.

HENZE G. FENGLER R. HARTMANN R. KORNHUBER B. JANKA-SCHAUB G. NIETHAMMER D AND RIEHM H. (1991). Six-year experience with a comprehensive approach to the treatment of recurrent childhood acute lymphoblastic leukemia (ALL-REZ BFM 85). A relapse study of the BFM group. *Blood*, **78**, 1166-1172.

HILL BT. DENNIS LY. LI X-T AND WHELAN RDH. (1985). Identification of anthracycline analogues with enhanced cytotoxicity and lack of cross-resistance to adriamycin using a series of mammalian cell lines in vitro. *Cancer Chemother. Pharmacol.*, **14**, 194-201.

HILL BT. HOSKING LK. SHELLARD SA AND WHELAN RDH. (1989). Comparative effectiveness of mitoxantrone and doxorubicin in overcoming experimentally induced drug resistance in murine and human tumour cell lines in vitro. *Cancer Chemother. Pharmacol.*, **23**, 140-144.

KASPERS GJL. PIETERS R. VAN ZANTWIJK CH. DE LAAT PAJM. DE WAAL FC. VAN WERING ER AND VEERMAN AJP. (1991). In vitro drug sensitivity of normal peripheral blood lymphocytes and childhood leukaemic cells from bone marrow and peripheral blood. *Br. J. Cancer*, **64**, 469-474.

KASPERS GJL. VEERMAN AJP. PIETERS R. BROEKEMA GJ. HUISMANS DR. KAZEMIER KM. LOONEN AH. ROTTIER MMA. VAN ZANTWIJK CH. HÄHLEN K AND VAN WERING ER. (1994). Mononuclear cells contaminating acute lymphoblastic leukaemic samples tested for cellular drug resistance using the methyl-thiazol-tetrazolium assay. *Br. J. Cancer*, **70**, 1047-1052.

KLUMPER E. PIETERS R. KASPERS GJL. LOONEN AH. HUISMANS DR. VAN ZANTWIJK CH. HÄHLEN K. VAN WERING ER. HENZE G AND VEERMAN AJP. (1993). Cytostatic drug resistance in childhood relapsed acute lymphoblastic leukemia. In *Acute leukemias*. Vol. IV. *Prognostic factors*. T Büchner (ed.) pp. 457-461. Springer: Berlin.

MADON E. GRAZIA G. DE BERNARDI B. COMELLI A. CARLI M. SAINATI L. PAOLUCCI G. CANINO R. COLELLA R. BAGNULO S AND DI PETRO N. (1987). Phase II study of idarubicin administered Iv to pediatric patients with acute lymphoblastic leukemia. *Cancer Treat. Rep.*, **71**, 855-856.

MIZUNO Y. HARA T. NAGATA M. TAWA A. TSURUO T AND UEDA K. (1991). Detection of multidrug-resistant protein, p-glycoprotein in childhood leukaemia and lymphoma. *Eur. J. Pediatr.*, **150**, 416-418.

MUGGIA FM AND GREEN MD. (1991). New anthracycline antitumor antibiotics. *Crit. Rev. Oncol. Hematol.*, **11**, 43-64.

NIEMEYER CM. REITER A. RIEHM H. DONNELLY M. GELBER RD AND SALLAN SE. (1991). Comparative results of two intensive treatment programs for childhood acute lymphoblastic leukemia: the Berlin-Frankfurt-Münster and Dana-Farber cancer institute protocols. *Ann. Oncol.*, **2**, 745-749.

PIETERS R. LOONEN AH. HUISMANS DR. BROEKEMA GJ. DIRVEN MWJ. HEYENBROK MW. HÄHLEN K AND VEERMAN AJP. (1990). In vitro drug sensitivity of cells from children with leukemia using the MTT assay with improved culture conditions. *Blood*, **76**, 2327-2336.

PIETERS R. HUISMANS DR. LOONEN AH. HÄHLEN K. VAN DER DOES-VAN DEN BERG A. VAN WERING ER AND VEERMAN AJP. (1991). Relation of cellular drug resistance to long-term clinical outcome in childhood acute lymphoblastic leukaemia. *Lancet*, **338**, 399-403.

PIETERS R. HONGO T. LOONEN AH. HUISMANS DR. BROXTERMAN HJ. HAHLEN K AND VEERMAN AJP. (1992). Different types of non-P-glycoprotein mediated multiple drug resistance in children with relapsed acute lymphoblastic leukaemia. *Br. J. Cancer*, **65**, 691-697.

PUI C-H. DE GRAAF SSN. DOW LW. RODMAN JH. EVANS WE. ALPERT BS AND MURPHY SB. (1988). Phase I clinical trial of orally administered 4-demethoxydaunorubicin (idarubicin) with pharmacokinetic and in vitro drug sensitivity testing in children with refractory leukemia. *Cancer Res.*, **48**, 5348-5352.

REID JM. PENDERGRASS TW. KRAILO MD. HAMMOND GD AND AMES MM. (1990). Plasma pharmacokinetics and cerebrospinal fluid concentrations of idarubicin and idarubicinol in pediatric leukemia patients: a childrens cancer study group report. *Cancer Res.*, **50**, 6525-6528.

SCOTT CA. WESTMACOTT D. BROADHURST MJ. THOMAS GJ AND HALL MJ. (1986). 9-Alkyl anthracyclines. Absence of cross-resistance to adriamycin in human and murine cell cultures. *Br. J. Cancer*, **53**, 595-600.

SPETH PAJ. LINSSEN PCM. BOEZEMAN JBM. WESSELS HMC AND HAANEN C. (1987). Cellular and plasma adriamycin concentrations in long-term infusion therapy of leukemia patients. *Cancer Chemother. Pharmacol.*, **20**, 305-310.

SPETH PAJ. MINDERMAN H AND HAANEN C. (1989). Idarubicin v daunorubicin: preclinical and clinical pharmacokinetic studies. *Semin. Oncol.*, **16**, 2-9.

STARLING KA. MULNE AF. VATS TS. SCHOCH I AND DUKART G. (1985). Mitoxantrone in refractory acute leukemia in children: a phase I study. *Invest. New Drugs*, **3**, 191-195.

TAN CTC. HANCOCK C. STEINHERZ P. BACHA DM. STEINHERZ L. LUKS E. WINICK N. MEYERS P. MONDORA A. DANTIS E. NIEDZWIECKI D AND STEVENS Y-W. (1987). Phase I and clinical pharmacological study of 4-demethoxydaunorubicin (idarubicin) in children with advanced cancer. *Cancer Res.*, **47**, 2990-2995.

TAWA A. ISHIHARA S. YUMARA K. HARA J. INOUE M. MURAYAMA F. KAWAI S. FUJIMOTO T. NOBORI U. NISHIKAWA A. TSURUO T AND KAWAHA K. (1990). Expression of the multidrug-resistance gene in childhood leukemia. *Jpn. J. Pediatr. Hematol.*, **4**, 38-43.

UBEZIO P. LIMONTA M. D'INCALCI M. DAMIA G. MASERA G. GIUDICI G. WOLVERTON JS AND BECK WT. (1989). Failure to detect the P-glycoprotein multidrug resistant phenotype in cases of resistant childhood acute lymphocytic leukaemia. *Eur. J. Cancer Clin. Oncol.*, **25**, 1895-1899.

UNGERLEIDER RS. PRATT CB. VIETTI TJ. HOLCENBERG JS. KAMEN BA. GLAUBIGER DL AND COHEN LF. (1985). Phase I trial of mitoxantrone in children. *Cancer Treat. Rep.*, **69**, 403-407.

VAN BELLE SJP. DE PLANQUE MM. SMITH IE. VAN OOSTEROM AT. SCHOEMAKER TJ. DENEVE W AND McVIE JG. (1986). Pharmacokinetics of mitoxantrone in humans following single-agent infusion or intra-arterial injection therapy or combined-agent infusion therapy. *Cancer Chemother. Pharmacol.*, **18**, 27-32.

VAN ECHO DA. WHITACRE MY. AISNER J. APPLEFELD MM AND WIERNIK PH. (1982). Phase I trial of aclacinomycin A. *Cancer Treat. Rep.*, **66**, 1127-1132.

VIETTI TJ. STEUBER CP. KIM TH. HOLCENBERG J. KAMEN B. MURRAY E AND CAPIELLO V. (1983). Mitoxantrone in children with advanced malignant disease. In *New Anticanter Drugs: Mitoxantrone and Bisantrene*. Rozenzweig M. (ed.) pp. 93-102. Raven Press: New York.

WEISS RB. (1992). The anthracyclines: will we ever find a better doxorubicin? *Semin. Oncol.*, **19**, 670-686.

YAMADA K. NAKAMURA T. TSURUO T. KITAHARA T. MAEKAWA T. UZAKA Y. KURITA S. MASAOKA T. TAKAKU F. HIROTA Y. AMAKI I. OSAMURA S. ITO M. NAKANO N. OGURO M. INAGAKI J AND ONOZAWA K. (1980). A phase II study of aclacinomycin A in acute leukemia in adults. *Cancer Treat. Rev.*, **7**, 177-182.